# Improving High-Precision BDS-3 Satellite Orbit Prediction Using a Self-Attention-Enhanced Deep Learning Model

**DOI:** 10.3390/s25092844

**Published:** 2025-04-30

**Authors:** Shengda Xie, Jianwen Li, Jiawei Cai

**Affiliations:** School of Information and Electronic Engineering, Zhejiang University of Science and Technology, Hangzhou 310023, China; shengda.xie@zust.edu.cn (S.X.); jiawei.cai@zust.edu.cn (J.C.)

**Keywords:** GNSS, BDS-3, ultra-rapid orbit, orbit prediction, time series forecasting, deep learning

## Abstract

Precise Global Navigation Satellite System (GNSS) orbit prediction is critical for real-time positioning applications. Current orbit prediction accuracy for the BeiDou Navigation Satellite System-3 (BDS-3) exhibits a notable disparity compared to GPS and Galileo, with limited advancements from traditional dynamic modeling approaches. This study introduces a novel data-driven methodology, Sample Convolution and Interaction Network with Self-Attention (SCINet-SA), to augment dynamic methods and improve BDS-3 ultra-rapid orbit prediction. SCINet-SA leverages deep learning to model the temporal characteristics of orbit differences between BDS-3 ultra-rapid and final products. By training on historical orbit difference data, SCINet-SA predicts future discrepancies, facilitating the refinement of ultra-rapid orbit estimates. The incorporation of a self-attention mechanism within SCINet-SA enables the model to effectively capture long-range temporal dependencies, thereby enhancing long-term prediction capabilities and mitigating the latency associated with final product availability. Rigorous experimental evaluation demonstrates the superior performance of SCINet-SA in enhancing BDS-3 ultra-rapid orbit prediction accuracy relative to alternative deep learning models. Specifically, SCINet-SA achieved the highest average relative improvement (IMP) in 3D Root Mean Square (RMS) error across 1 d, 7 d, and 15 d prediction horizons, yielding improvements of 21.69%, 18.66%, and 15.42%, respectively. The observed IMP range spanned from 7.78% to 38.91% for 1 d, 4.34% to 35.96% for 7 d, and 1.68% to 31.13% for 15 d predictions, underscoring the efficacy of the proposed methodology in advancing BDS-3 orbit prediction accuracy.

## 1. Introduction

The high-precision real-time positioning of the Global Navigation Satellite System (GNSS) is crucial for applications such as autonomous driving [1,2], geological hazard monitoring [3,4], and precision agriculture [5]. Accurate satellite orbit prediction is an essential part of providing high-quality GNSS positioning services. Common high-precision satellite orbit prediction products include GNSS ultra-rapid orbits, which are calculated using observation data collected over a short period [6]. Compared to final precise orbit products [7], GNSS ultra-rapid orbits offer higher timeliness. For the BeiDou Navigation Satellite System (BDS), the ultra-rapid orbit products of BDS-3 exhibit higher accuracy than those of BDS-2. However, there remains a gap in accuracy when compared to GPS and Galileo [8].

In recent years, considerable research has been dedicated to investigating the yaw attitude model [9,10] and solar radiation pressure model [11,12] of BeiDou satellites, which significantly influence orbit determination accuracy. However, despite these advancements, the precision of the refined dynamic models for BeiDou still lags behind that of GPS and Galileo.

In contrast to traditional physics-driven approaches, the studies presented in [13,14,15,16] offer an alternative approach to enhancing satellite orbit prediction accuracy, primarily focusing on refining Simplified General Perturbations Model 4 (SGP4) [17] predictions. By leveraging extensive historical Two-Line Orbital Element (TLE) data and employing the International Laser Ranging Service (ILRS) precise orbits as a benchmark, these studies quantify historical SGP4 propagation errors. These errors are then treated as observational data and modeled to predict future discrepancies. The predicted errors are subsequently employed to correct SGP4 predictions, thereby improving their predictive performance. These studies demonstrate the feasibility of data-driven methods for refining orbit predictions using historical data. However, traditional mathematical models, such as polynomial functions, exhibit limited representation capabilities, hindering their ability to capture the intricate patterns within the data.

Motivated by advancements in artificial intelligence (AI) techniques, researchers have introduced machine learning (ML) and deep learning (DL) models to enhance orbit prediction methods. These studies primarily employ Support Vector Machine (SVM) [18,19] and various neural network architectures, including Feed-Forward Neural Networks (FNNs) [20], Recurrent Neural Networks (RNNs) [21], and Long-Short Term Memory (LSTM) networks [22,23], as well as hybrid models [24]. By treating orbit error prediction as a time series forecasting [25] task, these approaches aim to refine the SGP4 model’s predictive accuracy. Additionally, some studies have extended these techniques to correct orbit prediction errors derived from broadcast ephemeris. By employing Convolutional Neural Networks (CNNs) [26] and Back Propagation (BP) neural networks [27], researchers have modeled historical orbit differences between the broadcast and precise ephemeris and incorporated these corrections into subsequent orbit predictions to improve overall accuracy. However, the aforementioned studies focus on satellite orbits with precisions ranging from tens of meters to kilometers. They do not investigate the feasibility of ML/DL algorithms in improving sub-meter orbit prediction accuracy.

In this study, we investigated the potential of data-driven deep learning algorithms to enhance the accuracy of BDS-3 real-time precise orbit products derived from dynamic models. To this end, a dataset spanning approximately 1000 days was constructed, encompassing the differences between BDS-3 ultra-rapid and final orbits within the radial, along-track, and cross-track (RTN) frame [28]. To facilitate long-term prediction of orbital difference data, we introduce Sample Convolution and Interaction Network with Self-Attention (SCINet-SA). This architecture is designed to extract multi-scale temporal features from historical orbital difference data, enabling the prediction of future discrepancies. Subsequently, these predicted residuals are utilized to refine ultra-rapid orbit predictions, effectively converging them toward the accuracy of final orbit solutions. Theoretically, real-time orbit correction is achievable, provided the prediction horizon exceeds the latency of the final precise orbit determination. The following sections first introduce the model structure and principles of SCINet-SA, followed by the workflow of using the deep learning model to improve the prediction portion of ultra-rapid orbit products. To verify the effectiveness of SCINet-SA, our innovative architecture designed for multi-scale temporal feature extraction, the experimental section evaluates its performance in improving ultra-rapid orbit prediction accuracy compared to various deep learning architectures.

## 2. Methodology

This study formulates the satellite orbit differences prediction problem as a time series forecasting task. Specifically, given a time series of orbit differences $X^{*}$ at epoch *t*, the objective is to predict the future τ epochs of orbit differences ${\hat{X}}_{t+1:t+\tau }=\left\{x_{t+1},\ldots ,x_{t+\tau }\right\}$ based on the past *T* epochs of orbit differences $X_{t-T+1:t}=\left\{x_{t-T+1},\cdots ,x_{t}\right\}$. Here, $x_{t}\in R^{d}$ represents the orbit differences vector at epoch *t*, and *d* denotes the number of dimensions in the orbit coordinates. For brevity, X and $\hat{X}$ will be used to represent the historical orbit differences time series and the predicted orbit differences time series, respectively.

### 2.1. SCINet-SA

**Figure 1:**
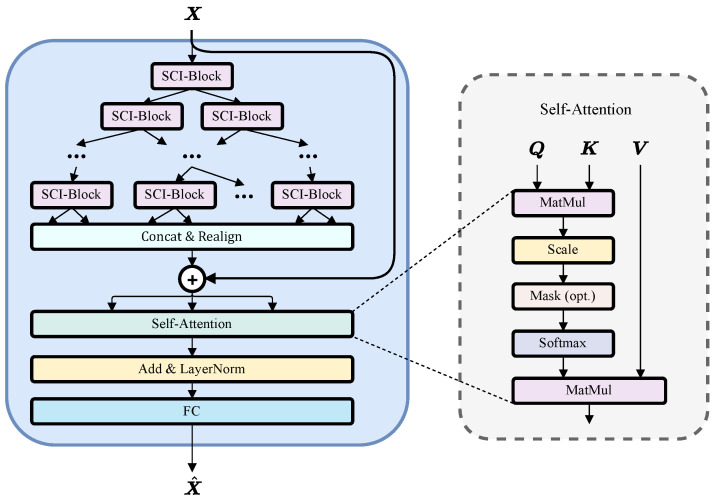
Overview of SCINet-SA architecture (**left**) and the implementation process of self-attention (**right**).

The proposed orbit differences prediction model, SCINet-SA (Figure 1), utilizes an encoder-decoder architecture [29]. The encoder integrates a SCINet module [30] and an attention module [31], while a fully connected (FC) layer serves as the decoder to output predictions.

SCINet, a hierarchical downsampling, convolutional, and interactive time series forecasting framework, effectively models time series with complex temporal dynamics. By iteratively extracting and exchanging information across multiple temporal resolutions, SCINet learns discriminative features that enhance predictability. SCINet was chosen as the foundational module due to its extensive evaluation on various real-world time series prediction datasets (ETTh [32], Traffic, Solar-Energy, Electricity, Exchange-Rate, and PeMS), where it consistently outperformed existing methods by a significant margin. Additionally, despite not explicitly modeling spatial relationships, SCINet demonstrates competitive performance on spatiotemporal sequence tasks.

To further strengthen the model’s ability to capture long-term dependencies and improve long-range forecasting, we incorporated a self-attention mechanism. Self-attention enables the model to compute the relevance of each position in a sequence with all other positions simultaneously. This mechanism allows the model to capture crucial relationships at different levels within the input sequence and can mitigate the impact of missing values in sparse time series data. Self-attention has been shown to enhance the performance of networks on time series tasks [33].

#### 2.1.1. SCINet Module

**Figure 2:**
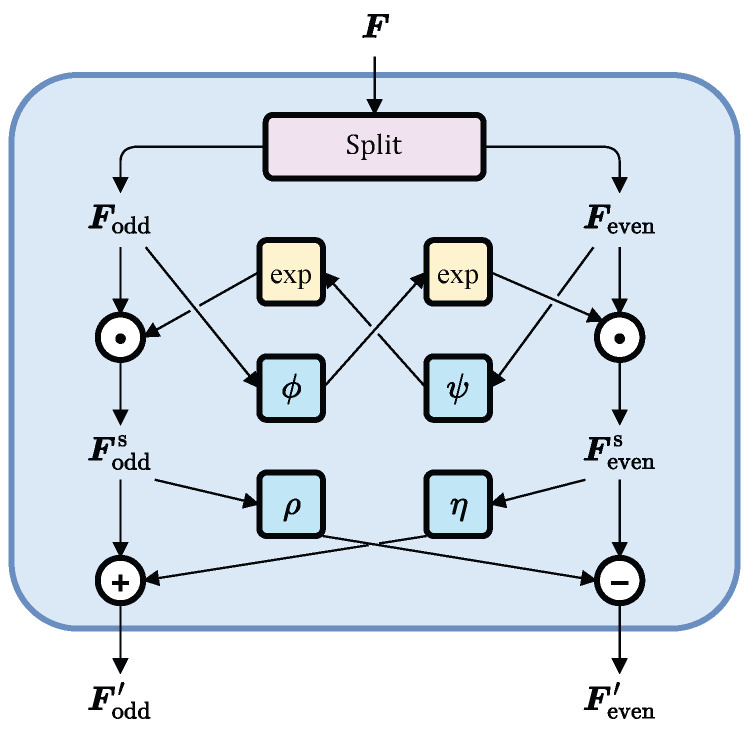
Structure of SCI-Block.

**Figure 3:**
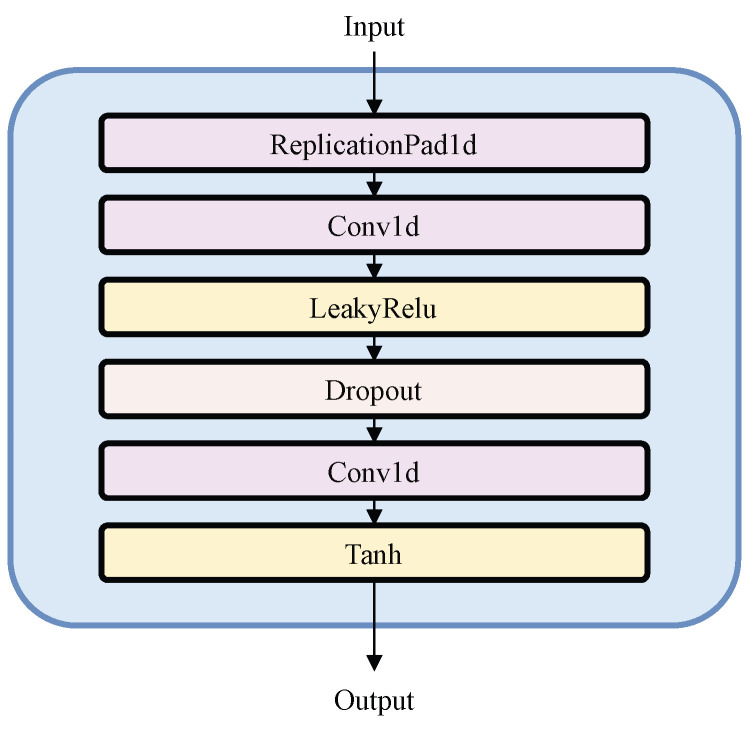
Details of ϕ, ψ, ρ, and η.

The core component of SCINet is SCI-Block. As illustrated in Figure 2, SCI-Block partitions the input feature sequence F into even and odd elements, downsampling them into two sub-sequences, $F_{\mathrm{even}}$ and $F_{\mathrm{odd}}$, which retain most of the original information but at a lower temporal resolution. These sub-sequences are then processed by a set of convolutional filters [34] (Figure 3). The filters ϕ, ψ, ρ, and η are independent, enabling the extraction of distinct yet valuable temporal features, thereby enhancing representational capacity. Subsequently, the sub-sequences undergo interactive learning to compensate for information loss during downsampling, yielding updated sub-feature sequences $F_{\mathrm{odd}}^{\prime }$ and $F_{\mathrm{even}}^{\prime }$, as described by the following equations:

(1) $$F_{\mathrm{odd}}^{s}=F_{\mathrm{odd}}⊙\exp\left(\phi (F_{\mathrm{even}})\right)$$

(2) $$F_{\mathrm{even}}^{s}=F_{\mathrm{even}}⊙\exp\left(\psi (F_{\mathrm{odd}})\right)$$

(3) $$F_{\mathrm{odd}}^{\prime }=F_{\mathrm{odd}}^{s}\pm \rho \left(F_{\mathrm{even}}^{s}\right)$$

(4) $$F_{\mathrm{even}}^{\prime }=F_{\mathrm{even}}^{s}\pm \eta \left(F_{\mathrm{odd}}^{s}\right)$$

where ⊙ denotes the Hadamard product, and exp represents the exponential function with base *e*.

The SCINet module comprises multiple SCI-Blocks arranged in a binary tree structure (with $2^{l-1}$ SCI-Blocks at the *l*-th level, where l=1,…,L and *L* is the total number of levels), as depicted in Figure 1. By processing the input sequence through SCI-Blocks at different levels, the module can learn effective features at various temporal resolutions. Information is progressively accumulated across multiple levels, such that deeper-level features incorporate higher temporal resolution information from lower levels. This mechanism facilitates the simultaneous capture of both short-term and long-term dependencies within the time series. Finally, all output sub-feature sequences are concatenated and rearranged back to their original order. This rearranged feature sequence is then added to the original time series through a residual connection, resulting in the final output feature S of the SCINet module.

#### 2.1.2. Attention Module

To enhance the model’s mid-to-long-term prediction capabilities, a self-attention mechanism was incorporated. Self-attention can be conceptualized as establishing interaction relationships among different vectors within a linear projection space of matrix S. This process is realized as follows:

The output of the SCINet module, denoted as $S=\left\{s_{1},\ldots ,s_{\tau }\right\}\in R^{d_{s}\times \tau }$, is linearly mapped into three distinct spaces, resulting in a query matrix Q, a key matrix K, and a value matrix V:

(5) $$Q=W^{q}S\;\left(W^{q}\in R^{d_{k}\times d_{s}},Q\in R^{d_{k}\times \tau }\right)$$

(6) $$K=W^{k}S\;\left(W^{k}\in R^{d_{k}\times d_{s}},K\in R^{d_{k}\times \tau }\right)$$

(7) $$V=W^{v}S\;\left(W^{v}\in R^{d_{v}\times d_{s}},V\in R^{d_{v}\times \tau }\right)$$

where W represents the weights (parameters) of the linear model, and $d_{q}$, $d_{k}$, and $d_{v}$ denote the dimensions of the query vector q∈Q, key vector k∈K, and value vector v∈V, respectively. Next, compute the attention distribution. For each query vector q, we employ a key-value pair attention mechanism. The attention distribution A is calculated as follows:

(8) $$A=\mathrm{softmax}\left(\frac{K^{⊤}Q}{\sqrt{d_{k}}}\right)$$

where the attention scores are computed using scaled dot-product attention. Due to the softmax function’s sensitivity to large or small inputs, scaling is performed using the hyperparameter $d_{k}$. Finally, we obtain the feature matrix B, which incorporates the attention scores across epochs, by performing a weighted summation based on the attention distribution A:

(9) B=VA

Following the self-attention mechanism, we employ residual connections [35] and layer normalization [36]. Residual connections address the vanishing and exploding gradient problems by directly adding the output of the previous layer to the output of the current layer while also facilitating faster model convergence during training. Layer normalization is then applied to normalize the sequence, which has been shown to significantly improve both the training speed and performance of the model.

### 2.2. Workflow of the Algorithm

**Figure 4:**
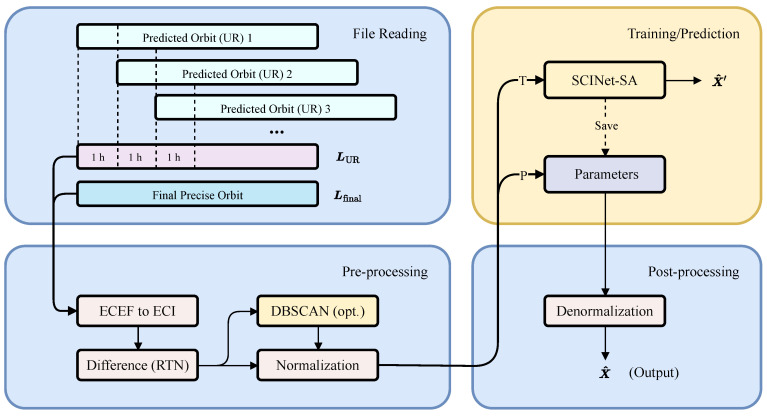
Workflow of the RTN differences prediction algorithm in this study.

The workflow for orbit differences prediction is illustrated in Figure 4. The entire process is divided into four distinct stages: (1) reading the ephemeris file, (2) data pre-processing, (3) model training or prediction, and (4) the post-processing of results.

#### 2.2.1. Ephemeris File Reading

We leveraged the ultra-rapid products from the Multi-GNSS Experiment (MGEX) network provided by the Wuhan University (WHU) International GNSS Service (IGS) Data Center [37]. These MGEX products offer the most comprehensive BDS ultra-rapid orbit data among all IGS Analysis Centers (ACs), with updates occurring every hour. As the model training utilizes continuous time series data, we concatenated the latest hour of data in epoch order to obtain the ultra-rapid predicted orbit sequence $L_{\mathrm{UR}}$. We then read the corresponding final orbit sequence $L_{\mathrm{final}}$ from the same AC, which was of the same length and epoch alignment (with an epoch interval of 15 min).

#### 2.2.2. Data Pre-Processing

The orbit data within the ephemeris file is presented in the Earth-Centered Earth-Fixed (ECEF) coordinate system, with its origin at the Earth’s center of mass and rotating with the Earth. This frame is not suitable for direct analysis of orbit differences. To facilitate this analysis, a transformation of the Earth-Centered Inertial (ECI) frame is necessary. Subsequently, differences between the ultra-rapid and final orbits were computed within the RTN frame. RTN is a local coordinate system that moves with the satellite, making it ideal for directly assessing position and velocity discrepancies in orbit. The radial (R) component signifies differences in the satellite’s orbital radius, the along-track (T) component captures along-track velocity and position deviations, and the cross-track (N) component reveals out-of-plane differences [28].

Taking into account the Earth Orientation Parameters (EOPs), the transformation from ECEF to ECI coordinates is expressed as follows [38,39]:

(10) $$r_{i}=R_{\mathrm{pn}}R_{\theta }R_{p}\cdot r_{e}$$

where $r_{i}$ and $r_{e}$ represent position vectors in the ECI and ECEF frames, respectively. The polar motion matrix $R_{p}$ and Earth rotation matrix $R_{\theta }$ are defined as

(11) $$R_{p}=\left[\begin{array}{ccc} \cos(x_{p}) & \sin\left(x_{p}\right)\sin\left(y_{p}\right) & \sin\left(x_{p}\right)\cos\left(y_{p}\right)\\ 0 & \cos(y_{p}) & -\sin(y_{p})\\ -\sin(x_{p}) & \cos\left(x_{p}\right)\sin\left(y_{p}\right) & \cos\left(x_{p}\right)\cos\left(y_{p}\right) \end{array}\right]$$

(12) $$R_{\theta }=\left[\begin{array}{ccc} \cos(\theta_{\mathrm{ERA}}) & \sin(\theta_{\mathrm{ERA}}) & 0\\ -\sin(\theta_{\mathrm{ERA}}) & \cos(\theta_{\mathrm{ERA}}) & 0\\ 0 & 0 & 1 \end{array}\right]$$

where $x_{p}$ and $y_{p}$ denote the components of polar motion in radians, and $\theta_{\mathrm{ERA}}$ is the Earth rotation angle derived from the difference between Universal Time (UT1) and Coordinated Universal Time (UTC). The precession and nutation corrections are encapsulated in the matrix $R_{\mathrm{pn}}$, which combines the precession matrix $R_{\mathrm{precession}}$ and the nutation matrix $R_{\mathrm{nutation}}$:

(13) $$R_{\mathrm{pn}}=R_{\mathrm{precession}}R_{\mathrm{nutation}}$$

where $R_{\mathrm{precession}}$ and $R_{\mathrm{nutation}}$ are derived from the precession angles (e.g., $\psi_{A}$, $\omega_{A}$, $\chi_{A}$) and nutation angles (e.g., Δψ, Δϵ), respectively. It should be noted that the computation of $R_{\mathrm{precession}}$ and $R_{\mathrm{nutation}}$ matrices involves detailed astronomical algorithms and data from the International Earth Rotation and Reference Systems Service (IERS) [40], utilizing series expansions and time-dependent parameters. By transforming $r_{e,\mathrm{UR}}\in L_{\mathrm{UR}}$ and $r_{e,\mathrm{final}}\in L_{\mathrm{final}}$ into ECI coordinates $r_{i,\mathrm{UR}}$ and $r_{i,\mathrm{final}}$, respectively, the orbit differences can be calculated as

(14) $$\Delta r_{i}=r_{i,\mathrm{UR}}-r_{i,\mathrm{final}}$$

The orbit differences were then transformed into the RTN frame to facilitate subsequent analysis. This transformation was achieved through the following steps [28]:

(15) $$e_{\mathrm{radial}}=\frac{r_{i,\mathrm{UR}}}{∥r_{i,\mathrm{UR}}∥}$$

(16) $$e_{\mathrm{along}}=\frac{r_{i,\mathrm{UR}}\times v_{i,\mathrm{UR}}}{∥r_{i,\mathrm{UR}}\times v_{i,\mathrm{UR}}∥}$$

(17) $$e_{\mathrm{cross}}=e_{\mathrm{radial}}\times e_{\mathrm{along}}$$

(18) $$R_{s}=\left[\begin{array}{ccc} e_{\mathrm{radial}} & e_{\mathrm{along}} & e_{\mathrm{cross}} \end{array}\right]$$

(19) $$\Delta r_{s}=R_{s}\cdot \Delta r_{i}$$

where $e_{\mathrm{radial}}$, $e_{\mathrm{along}}$, and $e_{\mathrm{cross}}$ represent the unit vectors in the RTN frame, $r_{i,\mathrm{UR}}$ and $v_{i,\mathrm{UR}}$ denote the position and velocity vectors in the ECI frame, respectively, and $\Delta r_{s}$ is the differences vector expressed in the RTN frame. Given that the original ultra-rapid products lacked velocity information, we derived the ECI velocities through numerical differentiation:

(20) $$v_{i,t}=\frac{r_{i,t+1}-r_{i,t-1}}{2\cdot \delta t}$$

where δt represents the time interval between consecutive epochs, set at 15 min in this study.

Potential outliers within the dataset were identified using the Density-Based Spatial Clustering of Applications with Noise (DBSCAN) algorithm [41]. DBSCAN, a density-based clustering method capable of discerning clusters of interconnected points with sufficiently high density, was employed to automatically detect anomalies in the RTN differences. A sliding window approach was implemented for outlier detection, with identified outliers replaced by the median value of the current window data. This optional step, applied judiciously when the number of outliers is minimal, mitigates the potential impact of outlier replacement on data distribution and subsequent model generalization.

Finally, Z-score normalization [42] was implemented to standardize the data to a uniform scale, facilitating model training. The Z-score formula is as follows:

(21) $$X_{s}=\frac{X_{o}-\mu }{\sigma }$$

In this context, $X_{o}$ and $X_{s}$ represent the original and standardized RTN differences, respectively. The symbols μ and σ denote the mean and standard deviation of $X_{o}$. This standardization process transforms the original RTN differences into a new sequence with a mean of zero and a standard deviation of one.

#### 2.2.3. Model Training/Prediction

Model training (T) and validation were performed using the orbit differences dataset derived from the pre-processed data. Given the distinct orbital elements of different satellites and, thus, the varying distributions of their orbit differences, individual models were trained for each BDS-3 satellite. This approach aimed to optimize the model’s adaptability to the unique characteristics of each satellite, thereby enhancing its predictive performance across the entire constellation.

For each satellite, 60% of its associated dataset was allocated for training. The model input consisted of historical orbit differences spanning *T* epochs, while the output was the predicted orbit differences for the subsequent τ epochs. During training, model parameters were updated using the Stochastic Gradient Descent (SGD) algorithm [43], and the optimal parameters were saved at each stage. A validation set, comprising 20% of the data, was used to monitor the model’s training progress. Early stopping was implemented if the validation error increased for three consecutive epochs relative to the preceding epoch. The model’s training performance was evaluated using Mean Absolute Error (MAE) [44] as the loss function. MAE quantifies the discrepancy between the predicted and actual orbit differences, as formulated below:

(22) $$L=\frac{1}{\tau }\sum_{t=1}^{\tau }\left|{\hat{x}}_{t}-x_{t}\right|$$

where ${\hat{x}}_{t}$ represents the output of the *t*-th epoch from the model, $x_{t}$ is the corresponding true orbit difference, and τ is the number of predicted epochs.

The prediction (P) process diverges from model training. During prediction, the model loads the optimal parameters saved during training, which are then frozen and no longer updated. Additionally, the model’s output must be denormalized to its original scale, yielding predicted orbit differences, denoted as $\hat{X}$. When $\hat{X}$ sufficiently approximates the actual orbit differences, the deviation of the ultra-rapid orbit relative to the final precise orbit can be corrected, thereby reducing the accuracy gap between the two.

## 3. Experiments

To evaluate the improvement in orbit prediction accuracy offered by SCINet-SA for all BDS-3 satellites in the WHU MGEX ultra-rapid ephemeris, we conducted experiments encompassing the satellites listed in Table 1. SCINet-SA’s hyperparameters are in Table A1. SCINet was chosen as a baseline to verify the effectiveness of our attention module. Additionally, we included several representative deep learning models in our analysis to demonstrate the superiority of our proposed method. These models include the following:

LSTM [45]: A refined RNN architecture designed to address the vanishing/exploding gradient problem often encountered during RNN training, particularly with long sequences. LSTM utilizes input, forget, and output gates to effectively manage information flow and capture long-term dependencies.

BiLSTM (Bidirectional LSTM) [46]: An extension of LSTM that processes sequences both forward and backward in time, enabling the model to leverage information from both the past and future for enhanced prediction.

SegRNN (Segment RNN) [47]: A specialized model for sequence segmentation tasks, dividing input sequences into segments and processing each with an RNN. SegRNN can share parameters between segments or utilize distinct parameters, and it incorporates a boundary detection mechanism to determine segment boundaries.

PRformer (Pyramidal Recurrent Transformer) [48]: A Transformer-based model designed for time series forecasting, addressing the challenge of temporal order representation. It incorporates Pyramid RNN Embeddings (PREs), which apply pyramidal one-dimensional convolutional layers to extract multi-scale convolutional features that preserve temporal structure. These features are subsequently processed by RNNs to capture hierarchical, sequence-sensitive representations. The resulting embeddings are fed into a standard Transformer encoder, enabling the model to effectively represent temporal dependencies, particularly in long lookback scenarios.

### 3.1. Model Evaluation Strategy

To assess the generalization ability of the models to data not encountered during training, 20% of the total epochs in the dataset were reserved as a test set. Model performance was quantified by calculating the Root Mean Square (RMS) differences between the original ultra-rapid orbits and the model-improved orbits, both relative to the final orbits in the RTN frame. Given the varying RMS values across different satellites, particularly between IGSO and MEO satellites, the relative improvement percentage was emphasized over absolute RMS improvements. The improvement (IMP) percentage represents the average percentage reduction in RMS achieved by the model-improved orbits compared to the original ultra-rapid orbits across all experiments for a single satellite. RMS and IMP are defined as follows:

(23) $${\mathrm{RMS}}_{\mathrm{UR}}=\sqrt{\frac{1}{\tau }\sum_{t=1}^{\tau }x_{t,\mathrm{UR}}^{2}}$$

(24) $${\mathrm{RMS}}_{\mathrm{model}}=\sqrt{\frac{1}{\tau }\sum_{t=1}^{\tau }{\left(x_{t,\mathrm{UR}}-{\hat{x}}_{t}\right)}^{2}}$$

(25) $$\mathrm{IMP}=\frac{{\mathrm{RMS}}_{\mathrm{UR}}-{\mathrm{RMS}}_{\mathrm{model}}}{{\mathrm{RMS}}_{\mathrm{UR}}}\times 100\%$$

where ${\mathrm{RMS}}_{\mathrm{UR}}$ and ${\mathrm{RMS}}_{\mathrm{model}}$ denote the RMS values of the ultra-rapid orbit and the model-improved orbit, respectively, relative to the final orbit. The term $x_{t,\mathrm{UR}}$ represents the orbit difference in a specific direction (radial, along-track, or cross-track) of the RTN frame for the ultra-rapid orbit at epoch *t*, while ${\hat{x}}_{t}$ represents the corresponding orbit difference predicted by the model at epoch *t*.

### 3.2. Results and Analyses

This section presents a quantitative and qualitative comparison of the performance of several deep learning models for ultra-rapid orbit prediction enhancement tasks across 27 different BDS-3 satellites. For SCINet-SA, we conducted a separate analysis focusing on two aspects: (a) the impact of different observation input window lengths on prediction performance and (b) the reliability of the algorithm when presented with a large amount of heterogeneous data. The experimental tasks tested include short-term prediction enhancement over 96 epochs (1 d) and medium-to-long-term prediction enhancement over 672 epochs (7 d) and 1440 epochs (15 d), which will be referred to as 1 d, 7 d, and 15 d prediction enhancements for brevity.

#### 3.2.1. Performance Comparison of SCINet-SA with Other Models

**Figure 5:**
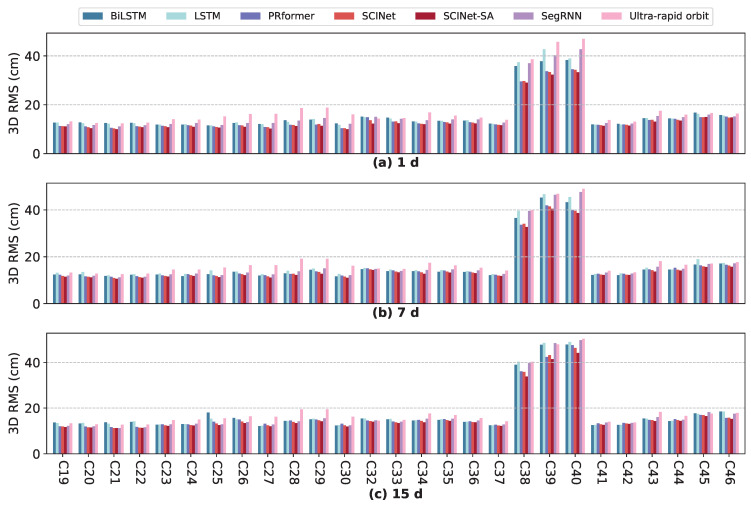
The 3D RMS of RTN differences between model-enhanced orbits and final orbits, with original ultra-rapid and final orbit differences as reference.

Figure 5 illustrates the comparison of 3D RMS values between the original ultra-rapid predicted orbits and the enhanced orbits after applying various models for 1 d, 7 d, and 15 d ultra-rapid prediction enhancements of BDS-3 satellites. After enhancement with SCINet-SA, the 3D RMS of all tested satellites decreased to varying degrees. The mean absolute improvements in 3D RMS for 1 d, 7 d, and 15 d orbit predictions were 4.41 cm, 3.58 cm, and 2.95 cm, respectively. It is evident that the original 3D RMS of the ultra-rapid orbits for the three IGSO satellites (C38, C39, C40) are significantly higher than those of the MEO satellites. Therefore, as discussed in Section 3.1, analyzing the relative improvement rate is more meaningful than the absolute improvement value in this study.

**Figure 6:**
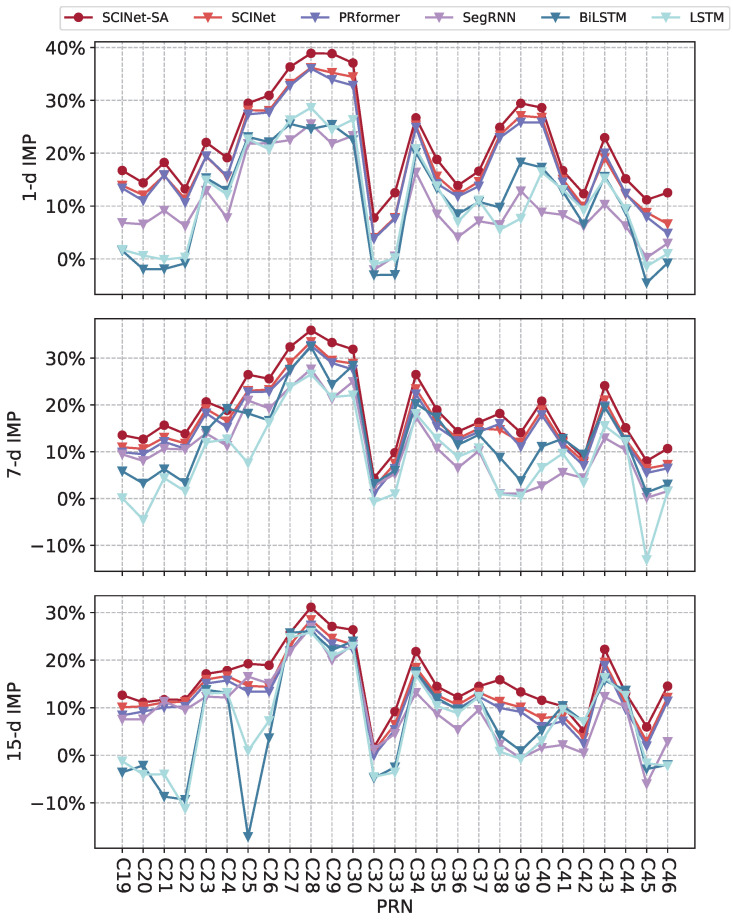
IMP (3D) of ultra-rapid orbit prediction for different satellites using SCINet-SA and other deep learning models.

Figure 6 compares the IMP (3D) of ultra-rapid orbit prediction for different prediction horizons using various models. Table 2 presents the average IMP results for all tested BDS-3 satellites using different models. The IMP (3D) of SCINet-SA for 1 d, 7 d, and 15 d prediction enhancements across all tested satellites are 7.78–38.91%, 4.34–35.96%, and 1.68–31.13%, respectively, with average IMP (3D) values reaching 21.69%, 18.66%, and 15.42%. SCINet-SA outperforms all other tested models, achieving superior improvement results for almost all tested satellites.

While SegRNN, LSTM, and BiLSTM demonstrate inconsistent improvement across different satellites, they can enhance orbit prediction for most satellites and even outperform SCINet-SA in certain tasks, such as the 15 d prediction enhancement for C42. However, their results are notably inferior to those of SCINet-SA for the majority of satellites. In some cases, their performance even degrades, resulting in negative optimization of orbit RMS, most evident in C25 and C45, which may be attributed to their limited capacity to capture complex temporal dependencies and spatial correlations present in satellite orbit data, especially when faced with irregularities or abrupt dynamic changes that are better modeled by architectures like SCINet-SA.

The SCINet architecture exhibited superior predictive accuracy compared to the recurrent neural network (RNN)-based models, namely SegRNN, LSTM, and BiLSTM. Furthermore, the integration of a self-attention module within SCINet-SA yielded a marginal but statistically significant enhancement in overall prediction performance, substantiating the efficacy of attention mechanisms in capturing relevant spatio-temporal dependencies within satellite orbit data. In contrast, the PRformer model, while demonstrating a performance level statistically superior to the aforementioned RNN architectures, exhibited a slight decrement in overall accuracy when compared to SCINet. However, it is noteworthy that for a subset of satellites, PRformer achieved comparable predictive accuracy to SCINet. These results suggest that PRformer possesses a degree of competence in modeling the complex, nonlinear patterns inherent in satellite orbital dynamics, albeit with potentially reduced robustness in certain orbital regimes.

#### 3.2.2. Analysis of RTN Differences Prediction Results

**Figure 7:**
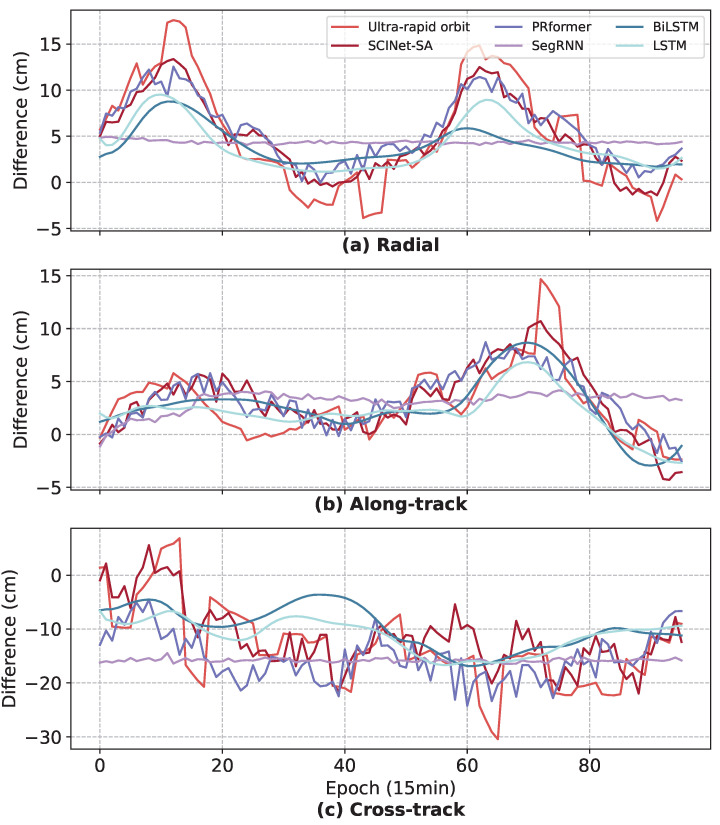
The 1 d (96 epochs) RTN differences prediction results for SCINet-SA and three RNN-based models.

**Figure 8:**
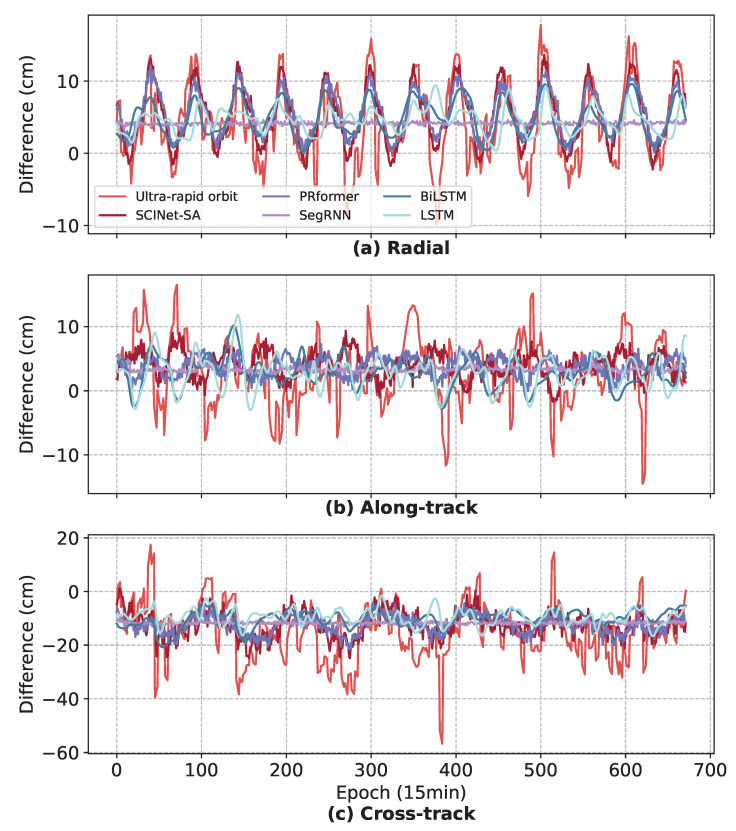
The 7 d (672 epochs) RTN differences prediction results for SCINet-SA and three RNN-based models.

**Figure 9:**
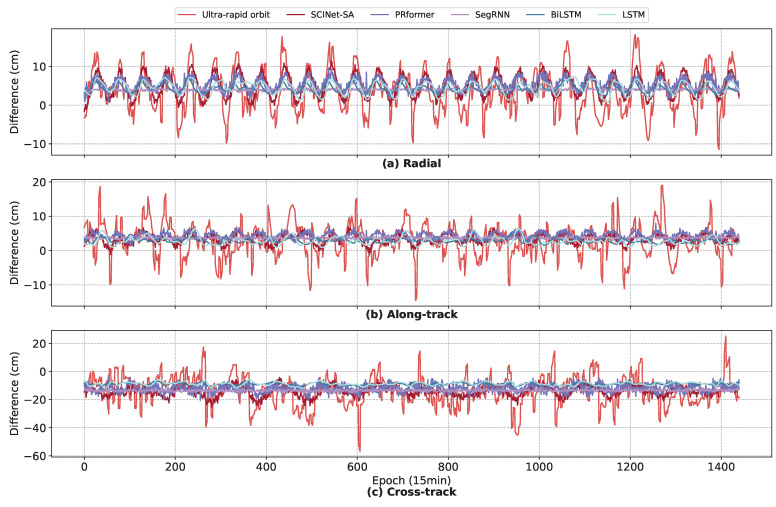
The 15 d (1440 epochs) RTN differences prediction results for SCINet-SA and three RNN-based models.

The preceding section demonstrated that the improvement results of SCINet-SA are superior to those of three RNN-based models (SegRNN, BiLSTM, and LSTM) and the Transformer-based PRformer. To investigate the reasons for this, we extracted the RTN difference prediction results of the five models on one instance of C28, as shown in Figure 7, Figure 8 and Figure 9. C28 was chosen because all models in this study achieved good improvement results for this satellite, making the comparison between models relatively fair.

As shown in Figure 7, in the 1 d short-term prediction, SCINet-SA, PRformer, LSTM, and BiLSTM fit the trend and fluctuations significantly better than SegRNN. However, for the high dynamic fluctuations in the data (Figure 7c, epochs 60–70), both LSTM and SegRNN exhibit severe underfitting. Although BiLSTM can capture it, the smoothed curve struggles to fit more complex fluctuations due to the limited representation capability of the model. Both SCINet-SA and SegRNN use a segmentation method for time series in their modeling mechanisms (although the segmentation methods differ), so theoretically, compared to sequence models (LSTM and BiLSTM), they have a stronger representation capability for the orbit difference data with complex fluctuations in this study. However, SegRNN is not sensitive to local fluctuations, which limits its short-term prediction performance. On the other hand, PRformer utilizes the self-attention mechanism to capture long-range dependencies within the sequence, thus exhibiting good performance in fitting the overall trend. However, as shown in Figure 7c, PRformer shows a slight deficiency in capturing high-frequency rapid changes, and its predicted curve is smoother than the true values. This may be because the global perspective of the self-attention mechanism can lead to a certain degree of information smoothing when dealing with local fine-grained temporal patterns. In contrast, SCINet-SA, through its structure combining the SCINet module and self-attention, can capture global trends while more finely modeling local fluctuations, thus demonstrating superior performance in short-term prediction.

In the relatively complex 7 d prediction task, the advantages of SCINet-SA in learning the local fluctuations and long-term trends of the data remain evident. The three RNN-based models all exhibit different problems. SegRNN still struggles to fit the local fluctuations in the data, while LSTM, due to its unidirectional sequential structure, has a relatively weak ability to capture the global dependencies of the time series. Therefore, for abnormal fluctuations in the data, such as epochs 350–480 in Figure 8a,c, LSTM shows poor robustness. BiLSTM, due to its bidirectional structure, has a stronger ability to model global dependencies and better robustness than LSTM, but noticeable anomalies still appear in the predictions when the data evolution patterns are too complex (Figure 8b, epochs 100–200). PRformer employs a structure combining Pyramid RNN Embeddings with a Transformer to effectively represent temporal dependencies, particularly in long lookback scenarios. As shown in Figure 8, it generally captures the overall trend well. However, over longer prediction horizons, PRformer tends to smooth out finer details. Specifically, in Figure 8b, compared to the true values, PRformer’s predictions exhibit a noticeable lag and reduced amplitude in capturing more rapid and intricate fluctuations, indicating that despite its strong long-range dependency modeling capabilities, it has certain limitations in handling highly complex local dynamics. This smoothing effect becomes more pronounced over the 7 d prediction window.

The superior performance of SCINet-SA can be attributed to the SCINet module’s enhanced incorporation of time series prior knowledge, enabling it to better capture both short-term (local temporal dynamics) and long-term (trends, periodicity) dependencies within the data compared to the other four models. Furthermore, SCINet-SA benefits from its self-attention mechanism, allowing for more effective capture of long-term latent patterns across the entire historical spatiotemporal data for improved future prediction. While PRformer also utilizes attention mechanisms, the SCINet module in SCINet-SA models finer-grained temporal relationships through its iterative sampling convolution and interaction process, enabling it to retain sensitivity to local dynamics even over longer prediction horizons.

The 15 d prediction task is more challenging due to the need to predict orbit differences for 1440 epochs. The performance of all tested models has declined to varying degrees (Table 2), with the most significant decline in Along-track direction prediction performance, where LSTM and BiLSTM even negatively optimize the orbit. As can be seen from Figure 9b, although SCINet-SA can capture the periodicity of time series to a certain extent, its ability to capture local fluctuations has declined significantly.

#### 3.2.3. Performance Comparison of SCINet-SA with Different Observation Windows

**Figure 10:**
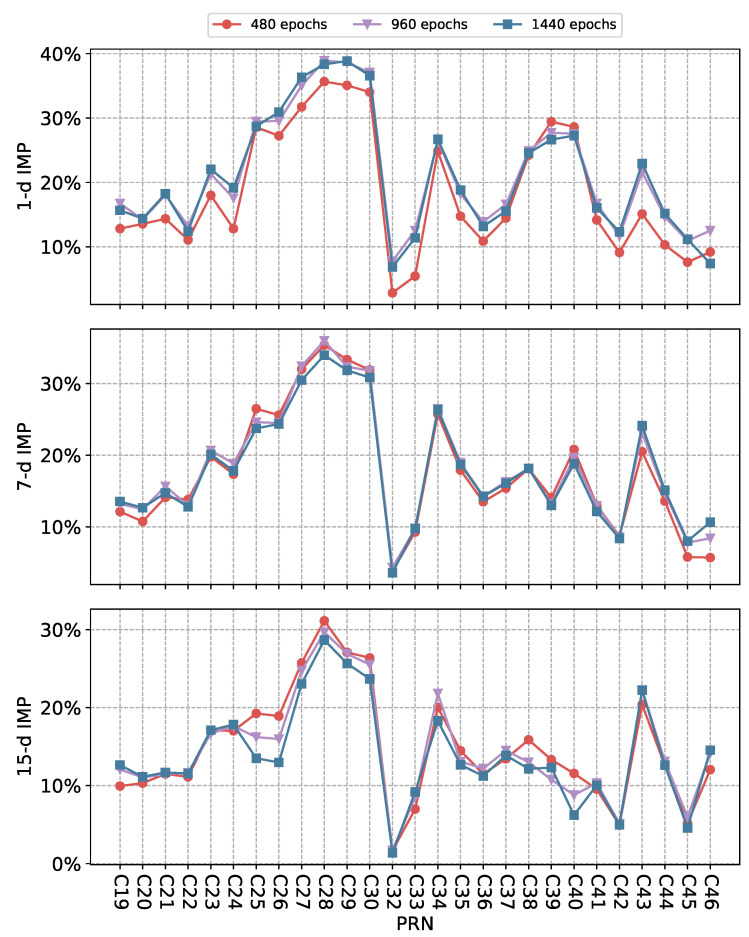
IMP (3D) of ultra-rapid orbit prediction for all tested satellites using SCINet-SA with different observation windows.

The deep learning-based orbit differences prediction methods in this study all use historical orbit differences as input observation data to predict subsequent data. To investigate the impact of observation window size on the ultra-rapid orbit prediction enhancement performance of SCINet-SA, experiments were conducted using input windows of 480, 960, and 1440 epochs, respectively. Figure 10 shows the IMP (3D) of SCINet-SA for different satellites in the 1 d, 7 d, and 15 d prediction enhancement tasks, and Table 3 presents the average IMP of all tested satellites. The performance of SCINet-SA for 1 d ultra-rapid orbit prediction enhancement is significantly affected by the input window, with the results of 960 and 1440 epoch input windows significantly outperforming 480 epochs on most satellites. Compared to the 480-epoch input window, the 960- and 1440-epoch input windows increased the average improvement rate of all tested satellites by 2.88% and 2.66%, respectively. However, in the 7 d and 15 d prediction tasks, the impact of the input window on improvement performance is not as significant as in the 1 d task, with the maximum fluctuation in IMP (3D) being only 0.72% (960 epochs input compared to 1440 epochs input in 15 d prediction).

This result can be attributed to the fact that in 1 d short-term prediction enhancement, having more historical data allows the model to more effectively capture the dependencies of orbit error data at multiple time resolutions. In the 7 d and 15 d prediction enhancements, since the input is the same type of data, the distribution of the data changes little within a certain time period, and SCINet-SA learns approximately repeated features, so increasing the window size does not have a significant impact on the prediction enhancement results.

#### 3.2.4. Reliability Analysis of SCINet-SA

The overall experimental results demonstrate that SCINet-SA can effectively improve the accuracy of BDS-3 ultra-rapid orbit prediction (Table 2). However, in practical applications, the reliability of a method is also a crucial indicator. To assess the reliability of SCINet-SA in improving ultra-rapid orbit prediction, we statistically analyzed all experimental results for 27 satellites across different prediction horizons.

**Figure 11:**
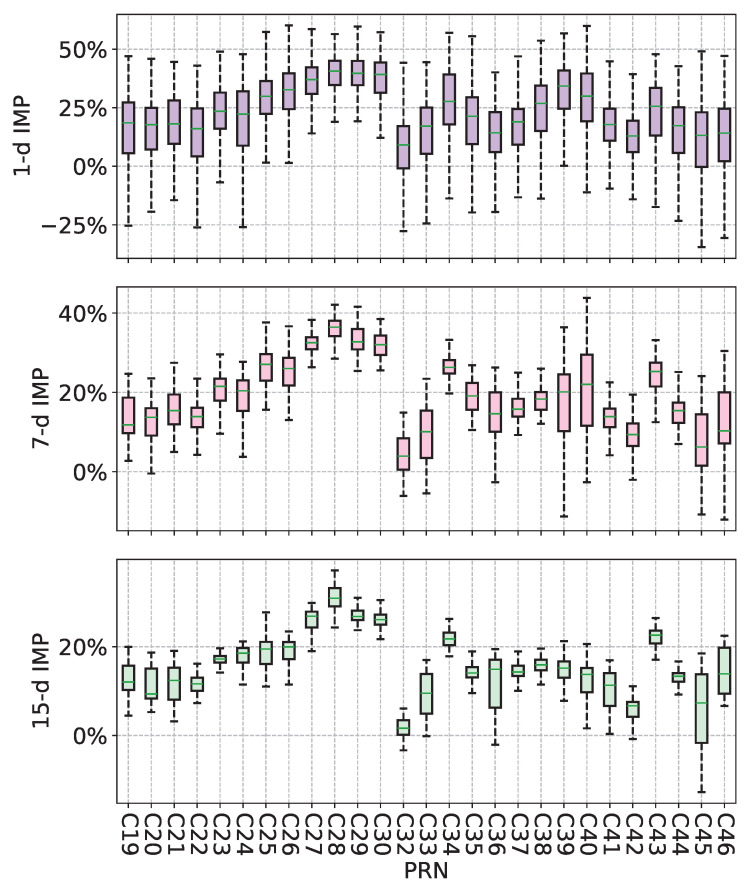
Distribution of IMP (3D) for all SCINet-SA experimental results.

Figure 11 presents the boxplots of IMP (3D) for all SCINet-SA experiments. In the improvement results across all prediction horizons, except for the 1 d and 15 d results of C32 and C45, the quartiles of all other satellites showed positive improvements. It is noteworthy that although the upper limit of the 1 d short-term prediction improvement results is higher, compared to the 7 d and 15 d results, its stability is poorer. Specifically, the box heights for the 1 d results of most satellites are larger, and most satellites exhibited cases of negative optimization.

Furthermore, as the prediction horizon increases (from 1 d to 15-d), the box heights of most satellites gradually decrease, indicating that the improvement rates become more concentrated, and the 15 d results demonstrate higher stability. This result highlights the effectiveness of the SCINet-SA model in long-term prediction improvements, particularly in the 15 d prediction range, where the model provides more stable and concentrated improvement effects.

## 4. Conclusions and Discussion

In this study, we propose SCINet-SA, a novel algorithm designed for long-term orbit difference prediction tasks. By integrating time series characteristics and attention mechanisms, SCINet-SA enhances the model’s ability to capture global temporal dependencies within orbit differences. The experimental results reveal the following key findings: (a) SCINet-SA significantly improves the prediction accuracy of BDS-3 ultra-rapid orbits, outperforming SCINet, PRformer, SegRNN, BiLSTM, and LSTM models. Specifically, for 1 d, 7 d, and 15 d ultra-rapid orbit predictions, the mean improvement percentages (IMPs) of SCINet-SA across 27 tested BDS-3 satellites are 21.69%, 18.66%, and 15.42%, respectively. (b) For long-term BDS-3 ultra-rapid orbit prediction improvement tasks, increasing the observation input window size does not significantly enhance performance. In 1 d short-term predictions, the IMP (3D) of SCINet-SA with 960- and 1440-epoch input windows is 2.88% and 2.66% higher than that with 480 epochs, respectively. However, for 7 d and 15 d predictions, the IMP (3D) differences among the three input windows are within 0.72%, indicating minimal impact.

Despite the strong performance of SCINet-SA in long-term predictions, this study has several limitations. First, in the 1 d short-term prediction, despite the higher upper limit of improvement results, the stability is poorer, and some satellites (e.g., C32 and C45) exhibit cases of negative optimization. This suggests that the model may have limited adaptability for certain satellites in short-term predictions, and further optimization is needed to enhance the stability of short-term forecasts. Second, although the concentration of improvement rates significantly increases as the prediction range extends (from 1 d to 15-d), this phenomenon may be partially attributed to the cumulative effect of errors in long-term predictions. Therefore, the model’s long-term improvement performance may rely to some extent on the smoothness of the data, and its adaptability to sudden changes requires further validation.

Finally, when deploying this model in actual GNSS services, we need to carefully consider its feasibility. From a preliminary perspective, while SCINet-SA has advantages in accuracy, its model complexity may introduce a certain computational burden. To meet the real-time requirements of GNSS services, future research will focus on analyzing the computational cost of the model, such as the number of model parameters and inference time, and exploring model lightweighting and acceleration strategies (e.g., model pruning and quantization) to achieve a balance between accuracy and efficiency. Simultaneously, regarding “expanding to other navigation systems (such as GPS and Galileo)”, we plan to collect and process ephemeris data from these systems and adjust the model structure and hyperparameters according to the characteristics of different systems. We recognize that the potential differences in orbital characteristics, data formats, and accuracy among different GNSS systems may require targeted model adjustments and additional computational resource investment. Future research will evaluate the model’s generalization ability on these systems and the associated computational costs.

## Figures and Tables

**Table 1:** BDS-3 satellite orbit data encompassed in this study.

PRN	C19	C20	C21	C22	C23	C24	C25	C26	C27
	C28	C29	C30	C32	C33	C34	C35	C36	C37
	C38 *	C39 *	C40 *	C41	C42	C43	C44	C45	C46
Time range	[9 April 2020 01:15, 4 January 2023 22:45]								
Total sample size	2,405,507								
Data partition	Training/Validation/Testing: 6/2/2								

Note: Pseudo-Random Noise (PRN) codes for Inclined Geosynchronous Orbit (IGSO) satellites, denoted with an asterisk (*). All other satellites listed are in Medium Earth Orbit (MEO).

**Table 2:** Average IMP (%) of ultra-rapid orbits for all tested satellites using SCINet-SA and other deep learning models at different prediction horizons (d).

Model	Horizon	Radial	Along-Track	Cross-Track	3D
SCINet-SA	1	29.77	19.14	15.22	21.69
	7	25.48	14.49	15.60	18.66
	15	20.73	8.88	13.54	15.42
SCINet	1	27.51	17.16	12.38	18.96
	7	22.98	12.20	13.41	16.28
	15	18.28	5.58	11.72	13.07
PRformer	1	26.77	16.62	11.78	18.39
	7	22.27	11.35	12.53	15.41
	15	17.23	4.52	10.74	12.02
SegRNN	1	12.33	12.93	5.69	10.49
	7	9.26	9.76	9.82	10.90
	15	7.24	5.47	8.99	9.32
BiLSTM	1	16.77	4.54	8.35	11.08
	7	19.76	4.55	12.80	13.12
	15	12.51	−10.35	10.46	6.82
LSTM	1	17.16	6.01	8.72	11.36
	7	11.05	0.18	9.37	8.59
	15	11.29	−6.20	9.82	7.16

**Table 3:** Average IMP (%) of ultra-rapid orbit prediction improvement for all tested satellites using SCINet-SA with different observation windows (epochs) at 1 d, 7 d, and 15 d prediction horizons.

Horizon	Window	Radial	Along-Track	Cross-Track	3D
1	480	27.27	17.15	11.51	18.37
	960	29.48	19.03	14.75	21.25
	1440	29.28	18.31	14.70	21.03
7	480	25.11	14.17	14.37	17.73
	960	25.08	13.72	15.35	18.22
	1440	24.79	12.71	15.24	17.93
15	480	20.80	8.66	12.63	14.78
	960	20.21	6.70	13.30	14.63
	1440	19.22	4.74	12.93	13.91

## Data Availability

The used WHU MGEX ultra-rapid and final orbit products can be found at ftp://igs.gnsswhu.cn/pub/gps/products/mgex/, accessed on 6 March 2025. The used EOPs canbe found at https://datacenter.iers.org/data/9/finals2000A.all, accessed on 6 March 2025.

## Abbreviations

The following abbreviations are used in this manuscript:

AC	Analysis Centers
AI	Artificial intelligence
BDS	BeiDou Navigation Satellite System
BiLSTM	Bidirectional Long Short-Term Memory
BP	Back Propagation
CNN	Convolutional Neural Networks
DBSCAN	Density-Based Spatial Clustering of Applications with Noise
DL	Deep learning
ECEF	Earth-Centered Earth-Fixed
ECI	Earth-Centered Inertial
EOP	Earth Orientation Parameters
FNN	Feed-Forward Neural Networks
GNSS	Global Navigation Satellite System
GPS	Global Positioning System
IERS	International Earth Rotation and Reference Systems Service
IGS	International GNSS Service
IGSO	Inclined Geosynchronous Orbit
ILRS	International Laser Ranging Service
IMP	Improvement
LSTM	Long-Short Term Memory
MAE	Mean Absolute Error
MEO	Medium Earth Orbit
MGEX	Multi-GNSS Experiment
ML	Machine learning
PRformer	Pyramidal Recurrent Transformer
PRN	Pseudo-Random Noise
RNN	Recurrent Neural Networks
RTN	Radial, Along-Track, Cross-Track
SCINet-SA	Sample Convolution and Interaction Network with Self-Attention
SGD	Stochastic Gradient Descent
SGP4	Simplified General Perturbations Model 4
SegRNN	Segment Recurrent Neural Network
SVM	Support Vector Machine
TLE	Two-Line Orbital Element
UT1	Universal Time
UTC	Coordinated Universal Time
WHU	Wuhan University
3D	Three-Dimensional

## Appendix A. Hyperparameters for SCINet-SA

Table A1 describes the main hyperparameters of SCINet-SA and their corresponding values used in the experimental results presented in this paper.

**Table A1:** SCINet-SA model hyperparameter description and experimental values.

Hyperparameter	Description	Value
level	The level count of SCI-Blocks	4
kernel	Convolution kernel size of convolutional filters	5
dilation	Whether to use dilation convolution	True
optimizer	Optimizer used	Adam
lr	Learning rate	$1\times 10^{-4}$
dropout	Dropout value in convolutional filters	0.5
loss	Loss function	MAE
n_head	Number of attention heads	1
batch_size	Batch size value	128
patience	Threshold for early stopping strategy	3
d_model	Input dimension of the attention module	3
input_len	Input length of SCINet-SA	480/960/1440
output_len	Output length of SCINet-SA	96/672/1440
